# New-onset of celiac disease during interferon-based therapy for hepatitis C

**DOI:** 10.1093/gastro/gou060

**Published:** 2014-09-11

**Authors:** Abhinav Vasudevan, John S. Lubel

**Affiliations:** ^1^Department of Gastroenterology, Eastern Health, Melbourne, Victoria, Australia and ^2^Eastern Health Clinical School, Monash University, Melbourne, Victoria, Australia

**Keywords:** interferon, celiac disease, hepatitis C

## Abstract

We present the case of a patient who first developed symptoms of celiac disease while on interferon-based therapy for treatment of chronic hepatitis C. He required hospital admission for symptom management and diagnostic work-up of severe diarrhoea. He made a rapid recovery with a gluten-free diet and was able to continue therapy. Consideration should be given to screening for celiac disease prior to the commencement of interferon-based therapy, particularly in high-prevalence populations.

## CASE PRESENTATION

A 50-year-old male presented to our clinic for management of chronic hepatitis C, found incidentally in the work-up for abnormal liver function tests during a recent inpatient stay for a bleeding gastric ulcer. He was a former intravenous drug user and had known of his hepatitis C status for 25 years but had never sought treatment. His other past history included depression, hypertension, previous inguinal hernia repair and ethanol excess and drinking up to 30 standard drinks per week. He made a complete recovery from his recent gastric ulcer, and 6-week follow-up gastroscopy showed resolution of the ulcer and no evidence of portal hypertension. At the time, duodenal biopsies were performed for possible duodenitis, but histology was normal with normal villous architecture. His medications were desvenlafaxine, pantoprazole, telmisartan and amlodipine.

Further tests showed his hepatitis C genotype was 1a, and the viral load was 1 949 000 IU/mL with an Interleukin-28B CC genotype. His initial blood tests showed a haemoglobin of 157 g/L (normal reference 130–170 g/L), white cells of 3.8 x 10^9^/L (4.0–10.0 x 10^9^/L), platelets of 181 x 10^9^/L (150–410 x 10^9^/L), bilirubin 7 μmol /L (<22 μmol/L), alanine aminotransferase (ALT) 285 IU/L (5–40 IU/L), γ-glutamyl transpeptadase (GGT) 694 IU/L (10–70 IU/L), alkaline phosphatase (ALP) 180 IU/L (30–120 IU/L) and albumin of 41 g/L (34–47 g/L). His international normalized ratio (INR) was 1.0 (0.9–1.2), ferritin 592 μg/L (30–400 μg/L) and transferrin saturations of 20% (15–45%). He underwent a fibroscan, which showed a median stiffness of 33.3 kilopascals with an interquartile range of 1.9, suggesting underlying cirrhosis.

He was commenced on bocepravir, interferon and ribavirin therapy for treatment of his hepatitis C, with an initial 4 weeks of pegylated interferon and ribavirin therapy, followed by 44 weeks of triple therapy. He weighed 83 kilograms and was started on 120 mg of PEGylated interferon weekly and 1200 mg of ribavirin, with 800 mg three times daily of bocepravir added later. Celiac antibodies were not tested prior to treatment and he did not have any symptoms of diarrhoea, bloating or weight loss. He was tolerating the regimen and had an undetectable viral load at Week 8 of therapy. During this week, however, he developed acute-onset watery diarrhoea, with greater than 20 bowel movements a day, with urgency and upper abdominal pain, associated with a weight loss of 11 kilograms. This continued for 3 weeks, so he presented to hospital for further work-up. On examination, he was apyrexial, there was mild epigastric tenderness without any guarding and no abdominal distension or organomegaly.

**Figure 1.**
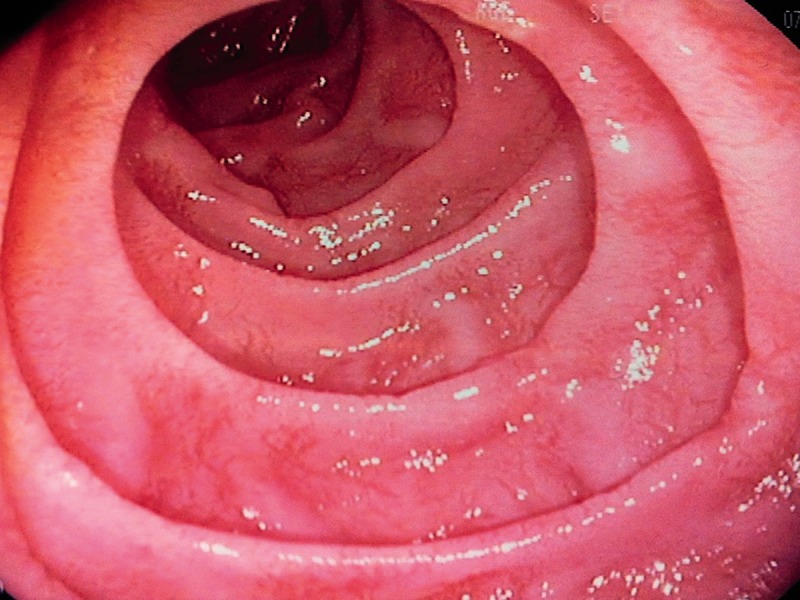
Endoscopic appearance of the second part of the duodenum. There are fissured folds with a scalloped appearance.

**Figure 2.**
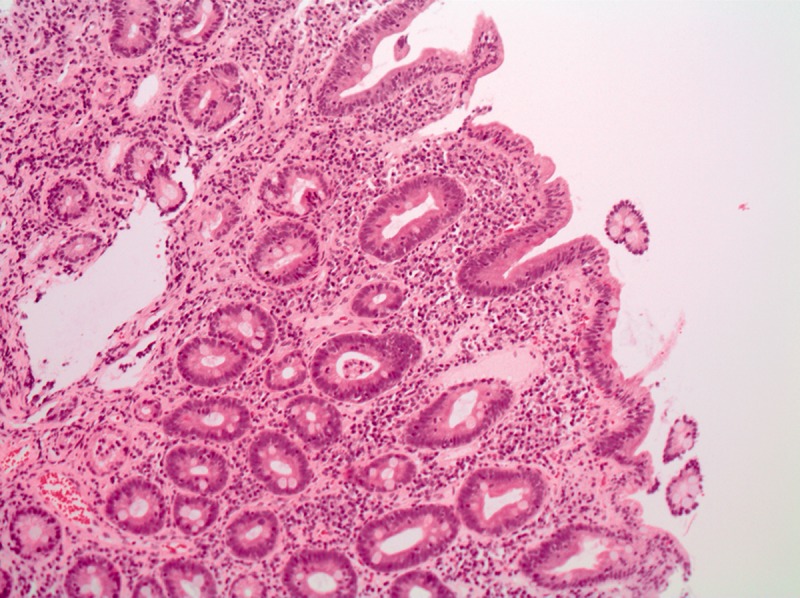
Histology of biopsy from the second part of the duodenum, showing villous atrophy.

His full blood count revealed a mild pancytopenia, with a haemoglobin of 125 g/L (normal reference 130–170 g/L), white cell count of 3.0 x 10^9^/L (4.0–10.0 x 10^9^/L), neutrophils 1.45 x 10^9^/L (2.00–7.00 x 10^9^/L) and platelets 91 x 10^9^/L (150–410 x 10^9^/L) with normal electrolytes, renal function and thyroid function. A stool sample contained no inflammatory cells and culture for bacteria and viruses was negative. Abdominal X-ray was normal. Colonoscopy was performed and biopsies of the colonic mucosa were normal but intra-epithelial lymphocytosis was noted in the terminal ileum. Gastroscopy demonstrated normal gastric mucosa, but the second part of the duodenum had fissured folds and a scalloped appearance (Figure 1). Histological examination of the duodenum showed moderate variable villous atrophy of the first and second parts of the duodenum, Marsh Classification 3b (Figure 2). The patient was tested for celiac-specific antibodies, which showed an antigliadin antibody (IgG) of 100 Units (normally <20) and a tissue transglutaminase antibody (IgA) of 54 Units (normally <20). Celiac HLA (human leukocyte antigen) genotyping confirmed that he was DQB1*02 (DQ2) positive and DQA1*05 positive. The patient was otherwise systemically well and was very keen to continue antiviral therapy. He was commenced on a gluten-free diet and experienced rapid resolution of symptoms. He is currently in the 28^th^ week of triple therapy and is tolerating it well, with minimal symptoms. His liver functions remained abnormal despite treatment of his celiac disease.

## DISCUSSION

PEGylated interferon is known to provoke autoimmune conditions through its immunomodulatory functions, which can lead to generalized immune cell activation and the development of autoantibodies [1]. It acts on the differentiation of TH2 to TH1 cells and improves the cytotoxicity of macrophages and natural killer cells [2]. Various autoimmune conditions have reportedly been unmasked during interferon therapy, including hyperthyroidism, autoimmune hepatitis, diabetes mellitus, interstitial pneumonitis, haemolytic anaemia and rheumatoid arthritis [3]. Ribavirin also increases TH1-mediated immune response while suppressing TH2 cells [4].

The release of interferon gamma and other cytokines, in response to exposure of the TH1 cells in the duodenal mucosa to ‘gluten' peptides, is also important for the development of celiac disease [5]. It is therefore plausible that interferon and ribavirin can unmask and cause *de novo* cases of celiac disease in a genetically susceptible host. However, population-based studies have showed mixed results regarding this association. In addition, a recent Italian study has suggested that, at a population level, interferon therapy does not appear to trigger celiac disease [6]. This may reflect a small study population or a low population prevalence of celiac disease. There have been several other similar cases reported in the literature, which all occurred while on interferon-based therapy [2, 7–10].

It has also been suggested that viral infections prime a mucosal T-cell response to gluten peptides. In addition, hepatitis C infections have been associated with the development of autoantibodies, including antiendomysial antibodies. Previous studies have attempted to determine whether there is an association between celiac disease and hepatitis C but none was found [11].

There has been previous debate about the utility of screening patients in high-prevalence populations for autoimmune conditions such as celiac disease prior to the commencement of interferon therapy [12]. A retrospective analysis of 534 cases showed a prevalence of raised transglutaminase antibodies amongst hepatitis C-positive individuals of 1.3%, compared with 0.4% in controls (no statistically significant difference) [13]. Among patients with elevated tranglutaminase antibodies, 86% of them had symptoms while on interferon therapy and 29% of these had to discontinue interferon treatment due to severe symptoms. In patients on interferon therapy who develop new symptoms, it is therefore important to have a high index of suspicion for reactivation of an autoimmune condition such as celiac disease. Celiac antibodies were not screened for in this gentleman prior to starting therapy. Had these been found, he could potentially have been started on a gluten-free diet prior to therapy, avoiding symptoms from celiac disease during his therapy.

Whilst interferon therapy in itself can cause diarrhoea in up to 10% of patients [14], it is important to exclude other causes—particularly infective and autoimmune—prior to attributing the symptoms to the interferon therapy. The symptoms of celiac disease when associated with interferon therapy may improve with a gluten-free diet and may not require the cessation of therapy [9].

Given the difficulty in determining the cause of new symptoms while on interferon-based therapy, baseline screening for celiac-associated antibodies prior to the commencement of therapy is beneficial in guiding further investigation and management in patients who develop symptoms that may be attributable to celiac disease during therapy. In addition, it helps to make patients fully aware of some of the potential adverse effects they may experience during therapy, given the high likelihood of activation in the setting of positive antibodies, and allow some patients to commence a gluten-free diet prior to commencing therapy.

**Conflict of interest:** none declared.
